# Early oral stepdown antibiotic therapy versus continuing intravenous therapy for uncomplicated Gram-negative bacteraemia (the INVEST trial): study protocol for a multicentre, randomised controlled, open-label, phase III, non-inferiority trial

**DOI:** 10.1186/s13063-022-06495-3

**Published:** 2022-07-19

**Authors:** I. Russel Lee, Steven Y. C. Tong, Joshua S. Davis, David L. Paterson, Sharifah F. Syed-Omar, Kwong Ran Peck, Doo Ryeon Chung, Graham S. Cooke, Eshele Anak Libau, Siti-Nabilah B. A. Rahman, Mihir P. Gandhi, Luming Shi, Shuwei Zheng, Jenna Chaung, Seow Yen Tan, Shirin Kalimuddin, Sophia Archuleta, David C. Lye

**Affiliations:** 1 National Centre for Infectious Diseases, Singapore, Singapore; 2 Department of Infectious Diseases, University of Melbourne, at the Peter Doherty Institute for Infection and Immunity, Melbourne, Victoria Australia; 3 School of Medicine and Public Health, Hunter Medical Research Institute, University of Newcastle, Newcastle, Australia; 4 University of Queensland Centre for Clinical Research, Royal Brisbane and Women’s Hospital Campus, Brisbane, Australia; 5 University Malaya Medical Centre, Kuala Lumpur, Malaysia; 6 Samsung Medical Center, Seoul, South Korea; 7 Department of Infectious Diseases, Imperial College London, London, UK; 8 Singapore Clinical Research Institute, Consortium for Clinical Research and Innovation, Singapore, Singapore; 9 Department of Infectious Disease, Sengkang General Hospital, Singapore, Singapore; 10 Division of Infectious Diseases, Ng Teng Fong General Hospital, Singapore, Singapore; 11 Department of Infectious Diseases, Changi General Hospital, Singapore, Singapore; 12 Department of Infectious Diseases, Singapore General Hospital, Singapore, Singapore; 13 Programme in Emerging Infectious Diseases, Duke-NUS Medical School, Singapore, Singapore; 14 Division of Infectious Diseases, Department of Medicine, National University Hospital, National University Health System, Singapore, Singapore; 15 Yong Loo Lin School of Medicine, National University of Singapore, Singapore, Singapore; 16 Lee Kong Chian School of Medicine, Nanyang Technological University, Singapore, Singapore; 17 Department of Infectious Diseases, Tan Tock Seng Hospital, Singapore, Singapore

**Keywords:** Gram-negative bacteraemia, Antibiotics, Early oral stepdown therapy, Oral fluoroquinolones, Oral trimethoprim-sulfamethoxazole, Health economic evaluation, Quality of life

## Abstract

**Background:**

The incidence of Gram-negative bacteraemia is rising globally and remains a major cause of morbidity and mortality. The majority of patients with Gram-negative bacteraemia initially receive intravenous (IV) antibiotic therapy. However, it remains unclear whether patients can step down to oral antibiotics after appropriate clinical response has been observed without compromising outcomes. Compared with IV therapy, oral therapy eliminates the risk of catheter-associated adverse events, enhances patient quality of life and reduces healthcare costs. As current management of Gram-negative bacteraemia entails a duration of IV therapy with limited evidence to guide oral conversion, we aim to evaluate the clinical efficacy and economic impact of early stepdown to oral antibiotics.

**Methods:**

This is an international, multicentre, randomised controlled, open-label, phase III, non-inferiority trial. To be eligible, adult participants must be clinically stable / non-critically ill inpatients with uncomplicated Gram-negative bacteraemia. Randomisation to the intervention or standard arms will be performed with 1:1 allocation ratio. Participants randomised to the intervention arm (within 72 h from index blood culture collection) will be immediately switched to an oral fluoroquinolone or trimethoprim-sulfamethoxazole. Participants randomised to the standard arm will continue to receive IV therapy for at least 24 h post-randomisation before clinical re-assessment and decision-making by the treating doctor. The recommended treatment duration is 7 days of active antibiotics (including empiric therapy), although treatment regimen may be longer than 7 days if clinically indicated. Primary outcome is 30-day all-cause mortality, and the key secondary outcome is health economic evaluation, including estimation of total healthcare cost as well as assessment of patient quality of life and number of quality-adjusted life years saved. Assuming a 30-day mortality of 8% in the standard and intervention arms, with 6% non-inferiority margin, the target sample size is 720 participants which provides 80% power with a one-sided 0.025 α-level after adjustment for 5% drop-out.

**Discussion:**

A finding of non-inferiority in efficacy of oral fluoroquinolones or trimethoprim-sulfamethoxazole versus IV standard of care antibiotics may hypothetically translate to wider adoption of a more cost-effective treatment strategy with better quality of life outcomes.

**Trial registration:**

ClinicalTrials.govNCT05199324. Registered 20 January 2022.

**Supplementary Information:**

The online version contains supplementary material available at 10.1186/s13063-022-06495-3.

## Administrative information

Note: the numbers in curly brackets in this protocol refer to SPIRIT checklist item numbers. The order of the items has been modified to group similar items (see http://www.equator-network.org/reporting-guidelines/spirit-2013-statement-defining-standard-protocol-items-for-clinical-trials/).

**Table:** 

Title {1}	Early oral stepdown antibiotic therapy versus continuing intravenous therapy for uncomplicated Gram-negative bacteraemia (the INVEST trial): Study protocol for a multicentre, randomised controlled, open-label, phase III, non-inferiority trial
Trial registration {2a and 2b}.	ClinicalTrials.gov identifier: NCT05199324
Protocol version {3}	Version 7.0, dated 27-May-2022
Funding {4}	Singapore’s National Research Foundation Central Gap Fund: Clinical Trials Grant – Investigator Initiated Trial (Award ID: CTGIIT19nov-0002)
Author details {5a}	I. Russel Lee^1^, Steven Y.C. Tong^2^, Joshua S. Davis^3^, David L. Paterson^4^, Sharifah F. Syed-Omar^5^, Kwong Ran Peck^6^, Doo Ryeon Chung^6^, Graham S. Cooke^7^, Eshele Anak Libau^1^, Siti-Nabilah B.A. Rahman^9^, Mihir P. Gandhi^9^, Luming Shi^9^, Shuwei Zheng^9^, Jenna Chaung^10^. Seow Yen Tan^11^, Shirin Kalimuddin^12, 13^, Sophia Archuleta^14, 15^, David C. Lye^1, 15–17^ ^1^ National Centre for Infectious Diseases, Singapore ^2^ Department of Infectious Diseases, University of Melbourne, at the Peter Doherty Institute for Infection and Immunity, Melbourne, Victoria, Australia ^3^ School of Medicine and Public Health, Hunter Medical Research Institute, University of Newcastle, Newcastle, Australia ^4^ University of Queensland Centre for Clinical Research, Royal Brisbane and Women’s Hospital Campus, Brisbane, Australia ^5^ University Malaya Medical Centre, Kuala Lumpur, Malaysia ^6^ Samsung Medical Center, Seoul South Korea ^7^ Department of Infectious Diseases, Imperial College London, London, United Kingdom ^8^ Singapore Clinical Research Institute, Consortium for Clinical Research and Innovation Singapore, Singapore ^9^ Department of Infectious Disease, Sengkang General Hospital, Singapore ^10^ Division of Infectious Diseases, Ng Teng Fong General Hospital, Singapore ^11^ Department of Infectious Diseases, Changi General Hospital, Singapore ^12^ Department of Infectious Diseases, Singapore General Hospital, Singapore ^13^ Programme in Emerging Infectious Diseases, Duke-NUS Medical School, Singapore ^14^ Division of Infectious Diseases, Department of Medicine, National University Hospital, National University Health System, Singapore ^15^ Yong Loo Lin School of Medicine, National University of Singapore, Singapore ^16^ Lee Kong Chian School of Medicine, Nanyang Technological University, Singapore ^17^ Department of Infectious Diseases, Tan Tock Seng Hospital, Singapore
Name and contact information for the trial sponsor {5b}	David C. Lye National Centre for Infectious Diseases, Tan Tock Seng Hospital, Singapore Email: david_lye@ncid.sg Phone: +65 6357 7457
Role of sponsor {5c}	David C. Lye is recipient of the Clinical Trials Grant – Investigator Initiated Trial (Award ID: CTGIIT19nov-0002) by Singapore’s National Research Foundation Central Gap Fund. David C. Lye has a role in the study design; collection, management, analysis, and interpretation of data; writing of reports; and decision to submit reports for publication. The grant funder has no role in the study design; collection, management, analysis, and interpretation of data; writing of reports; and decision to submit reports for publication.

## Introduction

### Background and rationale {6a}

The incidence of Gram-negative bacteraemia is rising globally and remains a major cause of morbidity and mortality. *Enterobacterales*, particularly *Escherichia coli* and *Klebsiella pneumoniae*, are the predominant pathogens isolated from blood [1, 2]. Although practice guidelines provide general recommendations for antibiotic treatment duration for Gram-negative bacteraemia, the optimal route of administration is yet to be definitively defined [3]. The majority of patients with Gram-negative bacteraemia initially receive intravenous (IV) antibiotic therapy. However, it remains unclear whether patients can step down to oral therapy after appropriate clinical response has been observed without compromising outcomes. Doctors must exercise judgement based on multiple factors such as severity of disease, host immune status, anticipated adherence and predicted adequacy of drug absorption and infection-site penetration. Limited data suggesting conversion to oral therapy is effective and safe are generally restricted to Gram-negative bacteraemia secondary to urinary tract infection [4–7]. Although the efficacy of early oral stepdown therapy versus continuing IV therapy for uncomplicated Gram-negative bacteraemia (not limited to urinary tract sources) is unknown, the advantages of oral therapy are apparent. Compared with IV therapy, oral therapy eliminates the risk of catheter-associated adverse events such as venous thrombosis, phlebitis, line breakage and catheter-associated bloodstream infections [8, 9]. Oral therapy enhances patient quality of life by eliminating discomfort associated with IV catheters, enabling mobility and reducing length of stay (LOS) in hospital [8, 9]. The healthcare cost of oral therapy is lower compared with IV therapy as there are no charges associated with placement/maintenance of central lines or drug preparation and administration [8, 10].

Tamma et al. recently compared patient outcomes from early oral stepdown therapy (within the first 5 days of treatment) versus continued IV therapy for monomicrobial *Enterobacterales* bacteraemia [7]. The retrospective multicentre study involved a 1:1 propensity score-matched cohort of 4967 unique cases. Key eligibility criteria included effective antibiotics administered from day 1 until treatment discontinuation, appropriate source control and clinical response by day 5. The authors found that 30-day all-cause mortality was not significantly different between 739 patients who received oral stepdown therapy versus 739 on continued IV therapy (HR 1.03, 95% CI 0.82–1.30). Patients who stepped down to oral therapy were discharged from hospital an average of 2 days earlier than those who continued IV therapy (5 [IQR 3–8] days vs. 7 [IQR 4–14] days; *p* < 0.001).

In an earlier retrospective single-centre study of *Enterobacterales* bacteraemia from the urinary tract, Rieger et al. compared outcomes between 135 patients who switched early to oral therapy (median 4 days of IV therapy) versus 106 patients who continued to receive IV therapy [4]. The key eligibility criterion was positive urine and blood cultures collected within 24 h with the same *Enterobacterales* pathogen. Treatment failure was not significantly different between patients who received IV antibiotics only versus those who received IV-oral antibiotics (3.8% [95% CI 1.0–9.4%] vs. 8.2% [95% CI 4.1–14.1%]; *p* = 0.19). Similar to Tamma et al. [7], the authors reported that patients who switched early to oral therapy were discharged from hospital approximately 2 days earlier than patients who continued to receive IV therapy (4.6 [IQR 3.1–7.8] days vs. 7.1 [IQR 4.0–17.5] days; *p* < 0.001).

To date, no randomised controlled trial (RCT) has been conducted to assess the efficacy of early oral stepdown therapy for uncomplicated Gram-negative bacteraemia that are not limited to urinary tract sources. Current management of uncomplicated Gram-negative bacteraemia entails a duration of IV antibiotic therapy with limited evidence from prospective studies to guide oral conversion.

### Objectives {7}

In this RCT, we aim to evaluate the clinical efficacy and economic impact of early stepdown to oral antibiotics versus continuing IV therapy for clinically stable / non-critically ill inpatients with uncomplicated Gram-negative bacteraemia.

We hypothesise that (a) early stepdown to oral antibiotics will be non-inferior to continuing IV antibiotic therapy in the primary outcome of 30-day all-cause mortality, and (b) early oral stepdown therapy will result in significantly lower health resource and service utilisation costs compared with continuing IV therapy.

### Trial design {8}

This study is designed as an international, multicentre, randomised controlled, open-label, phase III, non-inferiority trial with a non-inferiority margin of 6%. Eligible participants must be clinically stable / non-critically ill inpatients over the age of 18 years (≥21 years in Singapore) with uncomplicated Gram-negative bacteraemia. Randomisation to the intervention or standard arms will be performed with a 1:1 allocation ratio. Participants randomised to the intervention arm (within 72 h from the time of index blood culture collection) will be immediately switched to oral therapy. Participants randomised to the standard arm will continue to receive IV therapy for at least 24 h post-randomisation before clinical re-assessment and decision-making by the treating doctor. At the doctor’s discretion, patients in the standard arm may be switched to oral antibiotic therapy after continuing the IV therapy for at least 24 h post-randomisation. This data on oral antibiotic switch for patients in the standard arm will be recorded in the case report form (CRF). All study drugs (and dosage) would be those routinely used in clinical practice and will be ordered/dispensed from the hospital pharmacy as per site institutional practice. The recommended treatment duration is 7 days of active antibiotics (including empiric therapy), although treatment regimen may be longer than 7 days due to regimen extension or requirement for prolonged regimen as clinically indicated. Participants may be discharged home or to outpatient parenteral antibiotic therapy (OPAT) at any time post-randomisation according to the discretion of the treating doctor. At day 30 post-randomisation, participants will be assessed for the primary outcome of all-cause mortality. The key secondary outcome is health economic evaluation, including estimation of total healthcare cost (from healthcare system and patient perspective) to determine the economic impact of early oral stepdown therapy. Health services and resource utilisation cost data over the entire duration of the study will be collected from medical records or administrative sources whenever possible. A health outcome analysis will be conducted to assess patient quality of life and the number of quality-adjusted life years saved.

## Methods: participants, interventions and outcomes

### Study setting {9}

As Gram-negative bacteraemia can be seen in a large range of clinical situations spanning all hospital departments and patient populations, the study logically requires recruitment of patients from all areas of the hospital system. Gram-negative bacteraemias are usually treated in admitted patients and therefore inclusion of outpatients is not applicable. Recruitment in Singapore will be carried out in Tan Tock Seng Hospital, National University Hospital, Singapore General Hospital, Changi General Hospital, Ng Teng Fong General Hospital and Sengkang General Hospital. Recruitment will also be carried out in several overseas sites in Australia (Royal Brisbane and Women’s Hospital, Royal Melbourne Hospital), Malaysia (University Malaya Medical Centre), South Korea (Samsung Medical Centre) and UK (Imperial College Healthcare NHS Trust Hospital).

### Eligibility criteria {10}

Inclusion criteria will be non-restrictive allowing a representative and generalisable cohort of eligible participants including elderly patients:

1. ≥1 set of blood cultures positive for Gram-negative bacteria (GNB) associated with evidence of infection
2. Able to be randomised within 72 h of index blood culture collection
3. Age ≥18 years (≥21 years in Singapore)
4. Latest Pitt bacteraemia score <4
5. Patient or legal representative is able to provide informed consent

Exclusion criteria include:

1. Established uncontrolled focus of infection, including but not limited to:

- Undrained abdominal abscess, deep seated intra-abdominal infection and other unresolved abdominal sources requiring surgical intervention
- Central nervous system abscess (patients with focal neurology should have cranial CT prior to enrolment)
- Undrained moderate-to-severe hydronephrosis

2. Complicated infections, including but not limited to:

- Necrotising fasciitis
- Empyema
- Central nervous system infections and meningitis
- Endocarditis / endovascular infections

3. Sepsis as defined by infection with consequent acute organ dysfunction or septic shock as defined by systolic blood pressure <90 or mean arterial pressure <70 mmHg despite adequate fluid resuscitation
4. Polymicrobial bacteraemia involving Gram-positive pathogens or anaerobes (defined as either growth of ≥2 different microorganism species in the same blood culture, or growth of different species in ≥2 separate blood cultures within the same episode [<48 h] and with clinical or microbiological evidence of the same source)
5. Bacteraemia is due to a vascular catheter or intravascular materials (e.g. pacing wire, vascular graft) that cannot be removed
6. Specific Gram-negative pathogens that cannot be effectively treated with fluoroquinolones or trimethoprim-sulfamethoxazole, including but not limited to, *Burkholderia* spp. and *Brucella* spp.
7. Index GNB with resistance to fluoroquinolones AND trimethoprim-sulfamethoxazole
8. Hypersensitivity to fluoroquinolones AND sulphur drugs as defined by history of rash, urticaria, angioedema, bronchospasm, circulatory collapse or significant adverse reaction following prior administration
9. Unable to consume or absorb oral medications for any reason or unsuitable for ongoing IV therapy (e.g. no intravenous access)
10. Severely immunocompromised in the opinion of the treating doctor, including but not limited to, medical conditions such as:

- Active leukaemia or lymphoma
- Aplastic anaemia
- Bone marrow transplant within 2 years of transplantation or transplants of longer duration still on immunosuppressive drugs or with graft-versus-host disease
- Congenital immunodeficiency
- HIV/AIDS with CD4 lymphocyte count <200
- Neutropenia or expected post-chemotherapy neutropenia within 14 days from the time of screening, defined as absolute neutrophil count < 500 cells/μL

11. Women who are known to be pregnant or breast-feeding
12. Treatment is not with intent to cure the infection (i.e. palliative care)
13. Unable to collect patient’s follow-up data for at least 30 days post-randomisation for any reason
14. Treating doctor deems enrolment into the trial is not in the best interest of the patient
15. Previous enrolment in this trial

### Who will take informed consent? {26a}

The site principal investigator (PI) or his/her designee is responsible for ensuring freely given consent by each potential participant prior to the conduct of any protocol-specific procedures. The site PI may delegate the task of obtaining consent to appropriately qualified co-investigator(s). Consent must be documented by the participant’s dated signature on the informed consent form (ICF) together with the dated signature of the person conducting the consent discussion.

If the participant is illiterate or a translator is required, an impartial witness should be present during the entire consent discussion. Once the discussion has ended, the participant must sign and date the ICF, if capable. The impartial witness must also sign and date the ICF along with the person who conducts the consent discussion. If the participant does not have the capacity to consent, the written consent from a legal representative must be obtained. Capacity to consent should be assessed using the same process that is used when assessing consent capacity for treatment in the general hospital setting. This will take into account any potential legal authorities already in place and the patient’s baseline presentation. Capacity will be assessed in consultation with the treating team and the family if applicable. The investigator responsible for the consent process is responsible for ensuring the participant has the capacity to consent.

A copy of the signed and dated ICF together with the participant information sheet must be given to the participant prior to study participation. The participant or his/her legal representative must be informed in a timely manner of any new information that becomes available during the course of the study that may affect the participant’s willingness to continue study participation.

This study shall be conducted in accordance with the ethical principles laid out in the Declaration of Helsinki (most current issued version) and the National Statement on Ethical Conduct in Research Involving Humans (most current issued version).

### Additional consent provisions for collection and use of participant data and biological specimens {26b}

Not applicable. The protocols and its associated ICFs of substudies or ancillary studies from this trial, if any, will undergo an independent review by the relevant ethics committee (EC) / institutional review board (IRB).

## Interventions

### Explanation for the choice of comparators {6b}

High-bioavailability oral antibiotics such as fluoroquinolones or trimethoprim-sulfamethoxazole have been utilised for Gram-negative bacteraemia based on clinical and pharmacokinetics/pharmacodynamics (PK/PD) data [11–13]. However, the use of fluoroquinolones and trimethoprim-sulfamethoxazole increases the risk of potential adverse events (AEs) including *Clostridioides difficile*-associated diarrhoea [13–15]. As a result, interest has been spurred in the role of oral β-lactams that have low-to-moderate bioavailability. The question of whether oral β-lactams can be used as an efficacious alternative to oral fluoroquinolones or trimethoprim-sulfamethoxazole for Gram-negative bacteraemia is controversial.

Many doctors assume that patients who switch to oral fluoroquinolones or trimethoprim-sulfamethoxazole are less likely to experience treatment failure compared with patients who switch to oral β-lactams. This is due to the higher bioavailability and more favourable PK/PD profile of fluoroquinolones and trimethoprim-sulfamethoxazole compared with β-lactams. This assumption is supported by the retrospective study of Kutob et al., which investigated whether varying bioavailabilities of different oral antibiotics affected outcomes for Gram-negative bacteraemia predominantly from the urinary tract [16]. The authors found the risk of treatment failure was higher in patients who received antibiotics with low-to-moderate bioavailability compared to those who received antibiotics with high bioavailability.

However, in another retrospective study by Mercuro et al., clinical success was similar between patients who received oral fluoroquinolones and those who received oral β-lactams as stepdown therapy for *Enterobacterales* bacteraemia [17]. Likewise, in the earlier described study by Tamma et al., the authors found no difference in 30-day mortality between patients who switched to high-bioavailability agents versus those who switched to low-bioavailability agents [7]. It is noteworthy the studies by Kutob et al., Mercuro et al. and Tamma et al. were underpowered to determine whether bioavailability of oral antibiotics is crucial for successful treatment of Gram-negative bacteraemia [7, 16, 17].

In view of the uncertainty associated with high-bioavailability versus low-bioavailability agents for Gram-negative bacteraemia, the study team decided the oral stepdown arm of this RCT will consist only of fluoroquinolones or trimethoprim-sulfamethoxazole. This conservative decision (in not testing oral β-lactams) is supported by a recent review article highlighting many oral β-lactams have short half-life requiring frequent dosing that may negatively impact patient adherence [18]. Furthermore, the dose of oral β-lactams (unlike IV β-lactams) required to attain specific PD targets is still unclear and the determination of minimum inhibitory concentration of oral β-lactams is not routinely performed in many hospitals [18]. This conundrum is further complicated in clinical scenarios when dose adjustments are needed for patients with renal impairment [18]. A recent systematic review and meta-analysis revealed infection recurrence occurred more frequently in Gram-negative bacteraemic patients transitioned to oral β-lactams compared with oral fluoroquinolones, although all-cause mortality was not significantly different between the β-lactams group versus fluoroquinolones or trimethoprim-sulfamethoxazole group [19].

### Intervention description {11a}

The oral antibiotic options in the intervention arm of this RCT are fluoroquinolones (most commonly, ciprofloxacin) or trimethoprim-sulfamethoxazole. The recommended doses of oral antibiotics to be used for patients with normal renal function would be ciprofloxacin 750 mg twice daily (if body weight ≥70 kg) or ciprofloxacin 500 mg twice daily (if body weight <70 kg) or trimethoprim-sulfamethoxazole 5 mg/kg (for trimethoprim component) every 12 h up to a maximum trimethoprim-sulfamethoxazole (160 mg / 800 mg; double strength) two tablets twice daily. Doses may be adjusted in the setting of renal dysfunction according to the recommendations in Tables 1, 2 and 3.

**Table 1:** Recommended starting and maintenance doses of ciprofloxacin for patients with impaired renal function

Creatinine clearance (mL/min)	Dose of ciprofloxacin
>50	750 mg every 12 h (for patients <70 kg, dose at 500 mg every 12 h)
30–50	500 mg every 12 h
5–29	500 mg every 24 h
Haemodialysis or peritoneal dialysis	500 mg every 24 h (after dialysis)

**Table 2:** Recommended starting and maintenance doses of trimethoprim-sulfamethoxazole for patients with impaired renal function (weight-based adjustments)

Creatinine clearance (mL/min)	Dose of trimethoprim-sulfamethoxazole
>30	5 mg/kg (for trimethoprim component) every 12 h
15–30	2.5 mg/kg every 12 h
<15	2.5 mg/kg every 24 h
Haemodialysis or peritoneal dialysis	2.5 mg/kg every 24 h (after dialysis)

**Table 3:** Recommended starting and maintenance doses of trimethoprim-sulfamethoxazole for patients with impaired renal function (tablet-based adjustments)

Creatinine clearance (mL/min)	If usual recommended dose is 2 SS^a^ tablets (1 DS^b^ tablet) every 24 h or 3 times per week	If usual recommended dose is 2 SS tablets (1 DS tablet) every 12 h	If usual recommended dose is 4 SS tablets (2 DS tablets) every 12 h	If usual recommended dose is 4 SS tablets (2 DS tablets) every 8 h
>30	No dose adjustment	No dose adjustment	No dose adjustment	No dose adjustment
15–30	Reduce dose to ~50% of usual dose.	Reduce dose to ~50% of usual dose.	Reduce dose to ~50% of usual dose.	Reduce dose to ~50% of usual dose.
	Example: 1 SS tablet every 24 h or 3 times per week	Example: 2 SS tablets once, followed by 1 SS tablet every 12 h	Example: 2 SS tablets every 12 h	Example: 3 SS tablets every 12 h
<15	Reduce dose to ~25 to 50% of usual dose. Use with caution and appropriate monitoring.	Reduce dose to ~25 to 50% of usual dose. Use with caution and appropriate monitoring.	Reduce dose to ~25 to 50% of usual dose. Use with caution and appropriate monitoring.	Reduce dose to ~25 to 50% of usual dose. Use with caution and appropriate monitoring.
	Example: 1 SS tablet every 24 h or 3 times per week	Example: 2 SS tablets once, followed by 1 SS tablet every 12 or 24 h	Example: 2 SS tablets every 12 h OR 2 SS tablets once, followed by 1 SS tablet every 12 h	Example: 3 SS tablets every 12 h or 24 h

^a^ Abbreviation: *SS* single strength (trimethoprim-sulfamethoxazole 80 mg / 400 mg)

^b^ Abbreviation: *DS* double strength (trimethoprim-sulfamethoxazole 160 mg / 800 mg)

For patients in the standard arm, the IV antibiotic selection will be determined by the patient’s treating doctor based on his/her assessment of the ‘best available treatment’. The dosage, frequency and administration of study drugs (e.g. ceftriaxone 2 g daily, cefazolin 2 g three times daily) will also be determined by the treating doctor according to their hospital site’s clinical practice as well as consideration of patient’s renal function.

The study drugs are routinely used in clinical practice and will be ordered/dispensed from the hospital pharmacy as per site institutional practice. Study drugs will be stored and administered in accordance with standard pharmacy procedures. Storage conditions, temperature monitoring and accountability of the study drugs will be as per hospital pharmacy policy.

The recommended treatment duration for the intervention and standard arms is 7 days of active antibiotics (including empiric therapy), although treatment regimen may be longer than 7 days due to regimen extension or requirement for prolonged regimen as clinically indicated. Treatment duration is not the focus/subject of this trial as there are ongoing RCTs (e.g. BALANCE, NCT03005145) investigating optimal duration of antibiotic therapy for Gram-negative bacteraemia.

### Criteria for discontinuing or modifying allocated interventions {11b}

All the study drugs would be commonly used in clinical practice for Gram-negative pathogens. Study team will not be exposing participants to excess risk by study inclusion beyond the risks involved in standard therapeutic decisions and clinical management. The main risk for patients randomised to the intervention arm is that oral antibiotics may not be as efficacious compared with IV antibiotics for treatment of Gram-negative bacteraemia. In the event of microbiological or clinical failure of the oral antibiotic treatment, escalation to IV antibiotics may be initiated at the discretion of the treating doctor at any time point post-randomisation. Antibiotic escalation will not be considered a protocol deviation. Study team will be monitoring for any change in treatment strategy (e.g. switch to IV antibiotics from allocated oral antibiotics or vice versa) between the time of randomisation and day 30 due to (a) an AE deemed by the treating doctor to be of sufficient severity to change treatment strategy, or (b) presumed lack of efficacy of treatment strategy according to the judgement of treating doctor.

### Strategies to improve adherence to interventions {11c}

The participant’s drug charts (electronic and/or paper) will be reviewed by the study team for compliance with study treatment. Any missed dose(s) and non-study drugs administered will be recorded in the electronic case report forms (eCRFs).

An adherence check will be conducted at the end of the treatment regimen—pill count for participants on oral therapy and documentation of IV antibiotics administered for participants on IV therapy. Participants are deemed compliant if ≥90% of prescribed antibiotics were taken.

### Relevant concomitant care permitted or prohibited during the trial {11d}

Patients on ciprofloxacin may not take concomitant drugs that can cause prolongation of QT interval (e.g. class IA or class III antiarrhythmics).

### Provisions for post-trial care {30}

If participants follow the directions of the doctors and study team and are physically harmed due to the antibiotics or procedures given under the study plan, the hospital’s clinical trial compensation scheme will pay the medical expenses for the treatment of that injury. The participating hospital sites, without legal commitment, will compensate the participant for the injuries arising from his/her study participation without the participant having to prove the hospital is at fault. There are however conditions and limitations to the extent of compensation provided.

### Outcomes {12}

The primary outcome measure is to compare the all-cause mortality at day 30 post-randomisation in patients from the standard arm versus intervention arm.

The secondary outcomes measures are as follows:

1. All-cause mortality at days 14 and 90 from the time of randomisation
2. Duration of survival from the time of randomisation until day 90
3. Number of days on IV antibiotic therapy in the total index hospitalisation (including OPAT) for surviving participants from the time of randomisation until (i) hospital discharge and (ii) day 90
4. Number of days alive and free of antibiotics ((i) for all antibiotics and ii. for IV antibiotics) between the time of randomisation and day 90
5. AEs from the time of randomisation until day 90 including:

- *C. difficile*-associated diarrhoea
- Peripherally inserted central catheter and other central venous catheter complications (such as catheter-related bloodstream infection, catheter-related superficial or deep venous thrombosis/thrombophlebitis, catheter blockage and exit site infection) requiring line removal during index hospitalisation (including OPAT) from the time of randomisation
- Liver function test abnormalities or acute kidney injury

6. Change in treatment strategy (e.g. switch to IV antibiotics from allocated oral antibiotics or vice versa) between the time of randomisation and day 30 due to:

- An AE deemed by the treating doctor to be of sufficient severity to change treatment strategy
- Presumed lack of efficacy of treatment strategy according to the judgement of treating doctor

7. Time to being discharged alive from the total index hospitalisation (including OPAT and hospital in the home) between the time of randomisation and day 90 (note: any death occurrence within 90 days will be considered ‘90 days’)
8. Number of days alive and not in hospital (including OPAT) between the time of randomisation and day 90
9. Readmission or extended hospitalisation by day 90. Readmission is defined as a new hospitalisation for any cause occurring after discharge from the index hospitalisation. Extended hospitalisation is defined as >14 days of hospital LOS starting from the day of randomisation.
10. Health economic evaluation by day 90, including estimation of total healthcare cost from healthcare system and patient perspective
11. Assessment of patient quality of life via EQ-5D and WHOQoL-BREF on screening day, end of treatment day and day 90

Exploratory objective is Composite Desirability Of Outcome Ranking (DOOR) comprising:

- All-cause mortality at day 30 from the time of randomisation
- Clinical failure as defined by one or more of the following related to the index infection:
  - Extended duration of active antibiotics beyond 7–14 days, depending on planned duration of original regimen
  - Addition of a rescue antibiotic including switching to an alternate, non-study antibiotic
  - Additional unplanned therapeutic interventions
- Infectious complications as defined by one or more of the following related to the index infection:
  - Bloodstream relapse due to the same index GNB occurring any time between the completion of study drug intervention period and day 30
  - Distant seeding (i.e. growth of index GNB in a distant sterile site different from the original source of infection) occurring any time between completion of study drug intervention period and day 30
  - Local suppurative complication (e.g. renal abscess in pyelonephritis, empyema in pneumonia) that was not present at the time of randomisation and occurring any time between completion of study drug intervention period and day 30
- Presence of AEs or serious adverse events (SAEs) that lead to study drug discontinuation
- Quality of life by functional status, calculated as change from baseline functional bacteraemia outcome score (measured on the day of randomisation; Table 4) to functional bacteraemia outcome score measured on the last day of study drug treatment

**Table 4:** Functional bacteraemia outcome scoring system

7	Out of hospital; basically healthy; able to complete daily activities and has no healthcare interaction^*^ since discharge from the index hospitalisation in the last 7 days
6	Out of hospital; moderate signs or symptoms of disease; unable to complete daily activities OR has required 1–2 healthcare interactions* since discharge from the index hospitalisation over the last 7 days
5	Out of hospital; significant disability; requires a high level of care and assistance daily OR has required more than two healthcare interactions* since discharge from the index hospitalisation over the last 7 days
4	Hospitalised but not requiring stay in intensive care unit (ICU)
3	Hospitalised in ICU
2	Accommodated in a long-term ventilator unit
1	On palliative care in terminal phases of life (in hospital or at home)
0	Dead

^*^Healthcare interactions include home nursing visits, telehealth calls, emergency room visits and office visit

The scoring system for the composite DOOR outcome is presented in Table 5.

**Table 5:** Composite DOOR scoring system

Rank	Alive	How many of: 1. Clinical failure 2. Infectious complications 3. AEs or SAEs leading to study drug discontinuation	Quality of life
1	Yes	0 of 3	Tiebreaker based on QoL functional bacteremia outcome score
2	Yes	1 of 3	
3	Yes	2 of 3	
4	Yes	3 of 3	
5	No	Any	

Rank 1—Alive without any of the following binary (yes/no) components: (1) evidence of clinical failure, (2) an infectious complication or (3) any SAE or an AE leading to study drug discontinuation

Rank 2—Alive with one of the following binary (yes/no) components: (1) evidence of clinical failure, (2) an infectious complication or (3) any SAE or an AE leading to study drug discontinuation

Rank 3—Alive with two of the following binary (yes/no) components: (1) evidence of clinical failure, (2) an infectious complication or (3) any SAE or an AE leading to study drug discontinuation

Rank 4—Alive with all of the following binary (yes/no) components: (1) evidence of clinical failure, (2) an infectious complication or (3) any SAE or an AE leading to study drug discontinuation

### Participant timeline {13}

Table 6 shows the trial schedule of study activities.

**Table 6:** Trial schedule of study activities

Study activity	Screening	Antibiotic intervention			Follow-up			As necessary^b^
	−72 h to day 1	Days 1–7^a^	Before hospital discharge	End of treatment (window period: 3 days)	Day 14 (±3 days)	Day 30 (±3 days)	Day 90 (±3 days)	
Check eligibility	x							
Informed consent	x							
Demographics	x							
Charlson Comorbidity Index	x							
Physical examination, complication screening (if suspected)	x		x					x
Randomisation		x						
Study drug^a^		x						
Antibiotic history^c^	x			x^d^		x^d^	x^d^	
Blood cultures^e^								x
Full blood count^f^	x		x					x
C-reactive protein	x		x					x
Renal and liver panel^g^	x		x					x
Adherence check^h^				x^d^				
Adverse event monitoring^i^		x	x	x^d^	x^d,j^	x^d,j^	x^d,j^	
Review mortality status					x^d^	x^d^	x^d^	
Review for development of complications, relapse and distant seeding						x^d^		
Review hospital admission and discharge summaries					x^d^	x^d^	x^d^	
Review health service/ resource utilisation cost							x^d^	
Quality of life survey	x			x^d^			x^d^	

^a^Recommended duration of active antibiotic treatment (including empiric therapy) is 7 days. Final day of study treatment may be as early as day 4 considering the 72-h randomisation window, but will typically be between days 5 and 7. Regimen may be extended beyond 7 days if clinically indicated or treating doctor may prescribe a prolonged original regimen of >7 days according to his/her discretion

^b^According to the discretion of clinician if participant is still an inpatient

^c^Document all antibiotics taken during this bacteraemia episode including empiric treatment, study drug and any additional antibiotics administered

^d^Via telephone interview or home visit by the study team if participant has been discharged and information cannot be obtained via medical records or administrative sources

^e^Blood cultures usually ordered if patient is febrile >38 °C in the last 24 h during bacteraemia episode or if previous blood cultures remain positive or if any secondary infection is suspected

^f^Full blood count includes white blood cells, neutrophils, platelets and haemoglobin

^g^Renal panel includes sodium, potassium and creatinine; liver panel include alanine transaminase, aspartate transaminase, alkaline phosphatase and total bilirubin

^h^Pill count for participants on oral therapy and documentation of IV antibiotics administered for participants on IV therapy; participant deemed compliant if ≥90% of prescribed antibiotics taken

^i^Only AEs deemed by the treating doctor to be related to the study drug (from standard arm or intervention arm) will be documented in the CRF

^j^Only targeted AEs will be monitored during the follow-up time points, such as *C. difficile*-associated diarrhoea, catheter-related complications and liver and kidney function test abnormalities

### Sample size {14}

In an RCT comparing 7 days versus 14 days of antibiotic therapy for uncomplicated Gram-negative bacteraemia, 30-day all-cause mortality occurred in 4.9% of patients in the 7-day duration arm and 4.4% of patients in the 14-day duration arm [20]. In a retrospective multicentre study of propensity score-matched cohort with monomicrobial *Enterobacterales* bacteraemia, 30-day all-cause mortality was 13.1% for patients who received early oral stepdown therapy and 13.4% for those who continued to receive IV therapy [7]. Accurate estimation of mortality (for this study) is complicated by significant variability in reported mortality of past studies—likely influenced by geography and isolate resistance phenotype. We assumed 30-day mortality of 8% in the standard and intervention arms of this study—determined as the approximate mid-range from the two aforementioned studies [7, 20]. With a 6% non-inferiority margin, a total of 720 patients are needed to achieve 80% power with a one-sided 0.025 *α*-level after adjustment for 5% drop-out. Based on an expected mortality of 80% under a hypothetical situation where bacteraemic patients received no antibiotic treatment [21, 22], the standard arm treatment would have reduced mortality by 72% (from 80 to 8%). The pre-specified, 6% non-inferiority margin requires a 30-day mortality of ≤14% in the intervention arm, which preserves more than 90% of the 72% treatment effect of standard arm treatment to conclude non-inferiority. This is in accordance with requirements by the U.S. Food and Drug Administration (FDA) on non-inferiority margin to maintain at least 50% of treatment effect of the standard treatment.

### Recruitment {15}

Potential study participants will be identified on the basis of positive blood cultures by liaison between the investigators and the clinical microbiologists. No ‘cold-calling’ will be performed. The investigator will only approach the patient or his/her legal representative on invitation by the treating team (who will also have been notified of the blood culture results by the clinical microbiologist). The treating team will have the rationale of the study explained to them by the study investigator(s) before any patient contact occurs. On invitation by the treating team, the patient will be approached by a study team member to evaluate suitability for inclusion (by review of medical records and discussion with treating team) and have the study explained to him/her and be offered an opportunity to be enrolled.

## Assignment of interventions: allocation

### Sequence generation {16a}

Randomisation may occur if eligibility criteria have been met and informed consent has been obtained. Participants will be randomly assigned to either standard or intervention arms in a 1:1 ratio according to a randomisation list prepared in advance using a secure online randomisation system hosted by Singapore Clinical Research Institute. Randomisation will be stratified by country (Singapore, Australia, Malaysia, South Korea, UK) to ensure balance between study arms across countries.

### Concealment mechanism {16b}

Random sequence will be generated using random permuted blocks of unequal length. The day of randomisation is considered day 1 of treatment and the last dose of study drug to be given for the day is the dose next due prior to 23:59 h (i.e. the last scheduled dose prior to midnight).

### Implementation {16c}

Singapore Clinical Research Institute will generate the allocation sequence and assign participants to the treatment arms. The study team at the participating hospital sites in Singapore, Australia, Malaysia, South Korea and UK will enroll the participants.

## Assignment of interventions: blinding

### Who will be blinded {17a}

As the drugs in the standard and intervention arms have different routes of administration, this will be an open-label study.

### Procedure for unblinding if needed {17b}

Not applicable. This is an open-label study.

## Data collection and management

### Plans for assessment and collection of outcomes {18a}

The screening visit will include:

- Eligibility assessment (including a urine pregnancy test if applicable)
- Written informed consent
- Documentation of demographics
- Documentation of Charlson Comorbidity Index and antibiotic history since admission
- Physical examination including measurement of blood pressure and heart rate as well as cardiovascular, respiratory and abdominal examination
- Radiographic findings (if any)
- Baseline blood tests including full blood count (FBC; white blood cells, neutrophils, platelets, haemoglobin), C-reactive protein (CRP), renal panel (sodium, potassium, creatinine) and liver panel (alanine transaminase [ALT], aspartate transaminase [AST], alkaline phosphatase [ALP], total bilirubin [TBL])
- Blood culture results and antibiotic sensitivities of bacteraemia isolate
- EQ-5D and WHOQoL-BREF quality of life survey

Screening for metastatic complications will be undertaken if symptoms or examination findings are suggestive. Randomisation must be achieved within 72 h of the positive blood culture collection.

If participant is randomised to intervention arm, the first dose of the oral antibiotic will be administered by the ward nursing staff, and the participant may either remain as inpatient or be discharged to home. If participant is randomised to standard arm, administration of the IV ‘best available treatment’ antibiotic will continue for at least 24 h post-randomisation, which may be done as inpatient or in OPAT. The recommended treatment duration for both study arms is 7 days of active antibiotics (including empiric therapy), although treatment regimen may be longer than 7 days due to regimen extension or requirement for prolonged regimen as clinically indicated. Participants will be monitored by the clinical team in the hospital as per institutional practice until discharged, which typically entails standardised clinical assessments such as AE monitoring, physical examination and complication screening (if suspected). Blood cultures may be repeated on any day during the study period according to the discretion of the treating doctor to ensure clearance of bacteraemia especially if there is persistent fever or if previous blood cultures remain positive or if any secondary infection is suspected.

Before a participant is discharged from hospital, the standardised clinical assessments (physical examination, review of AEs, complication screening [at treating doctor’s discretion]) and blood tests (FBC, CRP, renal and liver panels) will be performed again.

At the end of study drug treatment (window period: 3 days), the study team will:

- Check for adherence to treatment regimen (pill count for participants in intervention arm and documentation of IV antibiotics administered for participants in standard arm)
- Review and document all antibiotics taken as well as AEs experienced since randomisation
- Request for completion of the quality of life survey

The above study procedures may be conducted via telephone interview or home visit by the study team if participant has been discharged from hospital and information cannot be obtained from medical records or administrative sources.

On day 14 (±3 days) and/or day 30 (±3 days), the study team will review and document:

- Mortality status of the participant (days 14 and 30)
- Specific AE occurrence, if any, since the last study review (days 14 and 30)
- Development of complications, relapse and distant seeding (day 30)
- Hospital admission and discharge summaries (days 14 and 30)
- Any antibiotics taken since the end of the study drug treatment regimen (day 30)

The above procedures may be conducted via telephone interview or home visit by the study team if participant has been discharged from hospital and information cannot be obtained from medical records or administrative sources.

On day 90 (±3 days), the study team will review and document:

- Mortality status of the participant
- Specific AE occurrence, if any, since the last study review
- Any antibiotics taken since the last study review
- Hospital admission and discharge summaries since the last study review
- Health services and resource utilisation cost data for the entire study duration

On day 90 (±3 days), the study team will also request for completion of the quality of life survey by the participant. The above procedures may be conducted via telephone interview or home visit by the study team if a participant has been discharged from hospital and information cannot be obtained from medical records or administrative sources.

### Plans to promote participant retention and complete follow-up {18b}

Participants or legal representatives have the right to choose to withdraw from the study at any time. The investigator may also discontinue a participant from the study or from treatment if deemed appropriate. The decision to withdraw a participant from the study must be discussed with the coordinating investigators. If a participant withdraws consent from study participation and withdraws consent for collection of future information, no further evaluations will be performed and no additional data will be collected. The study team may retain and continue to use any data or samples collected before such withdrawal of consent. Participants who abscond will continue to be followed, if possible, until the end of the trial to avoid missing data. Participants withdrawn from the treatment by the treating doctors will continue to be followed up to the end of the trial to avoid missing data and will be used in the intention-to-treat (ITT) analysis. Withdrawn participants will not be replaced. If a participant is withdrawn, the reason will be recorded in the database and source documents. Participants who deviate from intervention protocols, including premature discontinuation of study-related antibiotic therapy, will continue to have primary and secondary endpoint assessments for ITT analysis.

### Data management {19}

A trial database using the REDCap data management system will be developed with a secured web hosting facility. The eCRFs will collect clinical and laboratory-related information and will contain validation ranges for each variable to minimise data entry errors. The database will include information on demographics, underlying illnesses, antibiotic history, baseline and follow-up laboratory data including microbiologic data, and assessments of vital signs and AEs for the purpose of clinical outcome assessment. Data on hospital admission and discharge summaries will also be recorded. Source of bacteraemia, if known, will be noted. Trial data will be stored in a re-identifiable manner in the database using a unique screening number for each participant. For each potential participant screened (even those who are not eligible), the screening eCRF will be completed by the site PI or their delegate. For each participant enrolled, eCRFs will be completed. This also applies to records for those participants who fail to complete the study. The site PI will ensure the accuracy, completeness and timeliness of the data entered into the eCRFs and in all required reports. A comprehensive validation check programme will verify the data and automatically generate discrepancies for resolution by the investigator. Manual discrepancies can also be raised if necessary. The study team will manage the data and will conduct quality control of the data following their own standard operating procedures (SOP). Missing data or suspected errors will be raised as data queries (for example, by the lead study coordinator and monitor) and will be resolved prior to database lock and analysis. An audit trail will be maintained for tracking purposes. The clinical study report(s) and all analyses performed as well as the final data set will be archived together according to SOP.

### Confidentiality {27}

All study findings and documents will be regarded as confidential. The co-investigators and other study personnel must not disclose such information without prior written approval from the PI. Subject confidentiality will be strictly maintained to the extent possible under the law and local hospital policy. Identifiable information will be removed from any published data.

### Plans for collection, laboratory evaluation and storage of biological specimens for genetic or molecular analysis in this trial/future use {33}

An aliquot of the initial index blood culture isolate (as a suspension of pure bacterial colonies) will be stored at −80 °C in glycerol and nutrient broth or in Microbank™ at each hospital site microbiology laboratory as per standard practice. These bacterial isolates may be retrieved later for confirmatory susceptibility testing and genetic analysis for mechanisms of resistance. Any subsequent Gram-negative bacteraemia isolates that show resistance to the randomised antibiotic can be stored at the discretion of the site PI and microbiology laboratory, but is not mandated in the protocol.

## Statistical methods

### Statistical methods for primary and secondary outcomes {20a}

The primary analysis for this study will be performed on the modified intention-to-treat (mITT) population, which consists of all randomised patients but excludes those who withdraw consent prior to any post-randomisation assessment. Risk difference of 30-day all-cause mortality and its 95% confidence interval between standard and intervention arms will be estimated from a generalised linear model with binomial distribution and identity link function adjusted for country and other prognostic factors for the mITT population. If the upper limit of the 95% confidence interval falls below the 6% non-inferiority margin, non-inferiority will be concluded for early oral stepdown therapy relative to continuing IV therapy. As a special case of non-inferiority, if the upper limit of the 95% confidence interval falls below zero, superiority will be declared for early oral stepdown therapy. Supportive analysis of the primary endpoint will be performed with the per-protocol population, which consists of all randomised patients but excludes those who withdraw consent prior to any post-randomisation assessment and those who have a major protocol violation that may significantly affect the primary endpoint. Superiority of early oral stepdown therapy over continuing IV therapy will be evaluated with respect to outcomes such as LOS in hospital, as well as infection and health economic outcomes. Secondary outcome measures expressed as proportions will be compared between standard and intervention arms using chi-squared test, and risk difference as well as relative risk of the outcome measures will be calculated together with its 95% confidence interval. Mean difference and its 95% confidence interval will be provided for secondary outcome measures that are on interval scale, and comparison between study arms will be done using a two-sample *t*-test.

In addition to exploring the efficacy and safety of early oral stepdown therapy, this study will also analyse the economic viability of the proposed approach through a health economic evaluation according to ITT principle from healthcare system as well as patient perspectives. For base case analysis, private rate of each resource utilisation will be applied to calculate the average cost and savings. The health economic analysis will have two components:

1. If the RCT results confirm the non-inferiority of early oral stepdown therapy relative to continuing IV therapy, a cost saving analysis will be conducted to estimate the economic impact of the new treatment regimen;
2. If the RCT results fail to confirm the non-inferiority, a cost-effectiveness analysis will be conducted to estimate the incremental cost-effectiveness ratio (ICER) by comparing the difference in cost and clinical outcomes as well as quality of life between the two treatment arms (ICER=∆C/∆E).

To address possible variations in patient medical conditions and outcomes, a series of one-way sensitivity analyses will be conducted to address the potential impact of each parameter uncertainty and assess the robustness of study estimations for generalisability. Based on the sensitivity analysis, tornado plot will be generated to assess how much influence each of the variables has on the overall model. Additionally, probabilistic sensitivity analysis using the Monte Carlo simulation will be performed. Simulated results will be plotted in the cost-effectiveness plane to present the distribution of cost-effectiveness ratios. Cost-effectiveness acceptability curves will be generated to assess the probability variations of accepting each strategy by changing the threshold of willingness-to-pay.

### Interim analyses {21b}

An interim analysis, including efficacy and safety endpoints, will be performed after the first 50, 100 and 350 subjects have completed the 90-day study period or as determined by the Data Safety Monitoring Board (DSMB). A DSMB Charter detailing the required interim analysis will be prepared at the beginning of the trial. If there is a significant safety concern raised or the observed difference in proportion of patients reaching the primary endpoint exceeds the non-inferiority margin of 6%, the DSMB may recommend the trial should be stopped. The timing of additional interim analyses will be determined by the DSMB.

### Methods for additional analyses (e.g. subgroup analyses) {20b}

Subgroup analysis of the primary endpoint will also be performed, although the study power in subgroups may be low. The subgroups include (a) the urinary tract as source of infection, (b) all other sources of infection except the urinary tract, (c) index GNB that is multidrug-resistant and (d) index GNB that is non-multidrug-resistant. The definitions of multidrug resistance are below.

- Extended-spectrum beta-lactamase (ESBL) or AmpC-producing *Enterobacterales* isolates are considered multidrug-resistant. *Enterobacterales* demonstrating resistance to oxyimino-beta-lactam substrates (cefotaxime and ceftazidime) are considered ESBL or AmpC positive.
- *Pseudomonas* spp. isolates resistant to ≥3 of the following antimicrobial agents are considered multidrug-resistant: antipseudomonal penicillins (e.g. piperacillin), antipseudomonal cephalosporins (e.g. ceftazidime), fluoroquinolones (e.g. ciprofloxacin), carbapenems (e.g. imipenem, meropenem) and aminoglycosides.
- *Acinetobacter* spp. isolates resistant to ≥3 of the following antimicrobial agents are considered multidrug-resistant: imipenem (or meropenem), levofloxacin (or other fluoroquinolones), ceftazidime, colistin, tobramycin (or other aminoglycosides) and piperacillin–tazobactam.

### Methods in analysis to handle protocol non-adherence and any statistical methods to handle missing data {20c}

Participants may voluntarily withdraw their consent from study participation at any time and for any reason without penalty. A participant may also be withdrawn from participation in the study for the following reasons: (a) termination of study or (b) any new information becomes available that makes continuing participation unsafe. An early termination occurs when an enrolled participant withdraws consent to participate in the study, regardless of circumstances, prior to the primary outcome assessment at day 30. All participants terminating early from the study, regardless of cause, will be classified as non-evaluable and will be analysed as a ‘failure’ in the efficacy analyses. The reason(s) for early termination should be reflected in the source documentation and on the applicable eCRFs. In all cases, the reasons why a participant is withdrawn must be recorded in detail and entered into the eCRF. Patients who withdraw from the study will not be replaced. Patients whose randomised treatment is changed due to an AE or treatment failure or an unintentionally fulfilled exclusion criterion will remain in the study to assess outcomes. If the unintentionally fulfilled exclusion criterion/criteria is judged to be critical (i.e. it will significantly affect clinical outcomes), the case may be excluded from efficacy analysis although it will still be included in safety analysis regardless of study arm allocation.

### Plans to give access to the full protocol, participant-level data and statistical code {31c}

The dataset analysed during the current study and statistical code are available from the corresponding author on reasonable request, as is the full protocol.

## Oversight and monitoring

### Composition of the coordinating centre and trial steering committee {5d}

This trial will be managed by Singapore ID Clinical Research Network (SCRN), which was established in 2013 to coordinate multicentre clinical ID research in Singapore and internationally. SCRN is governed by an executive committee comprising clinical investigators from eight public hospitals in Singapore. SCRN is supported by project managers, clinical research fellows and clinical research coordinators based across multiple hospital sites in Singapore. The SCRN team will assist in the execution of multicentre legal agreements; application for EC/IRB and regulatory approvals; local participant enrolment and follow-up; data management, cleaning and analyses; and drafting of reports and manuscripts. At the time of writing (16-Mar-2022), the study’s overall PI is in the progress of forming a Global Trial Steering Committee (GTSC), which will be the key decision-making body for the trial. The roles and responsibilities of the GTSC include, but are not limited to, providing oversight to ensure the trial’s timeline, milestones and key performance indicators are met as well as proper execution of the trial in each of the participating sites in the different countries.

### Composition of the data monitoring committee, its role and reporting structure {21a}

A DSMB comprising two independent ID doctors and an independent statistician has been established. The primary roles of the DSMB are to (a) periodically review and evaluate the accumulated trial data for participant safety, efficacy of the intervention arm, as well as trial conduct and progress, and to (b) make recommendations regarding the continuation, modification or termination of the trial.

### Adverse event reporting and harms {22}

An AE is defined in the International Conference on Harmonisation-Good Clinical Practice (ICH-GCP) as ‘any untoward medical occurrence in a patient or clinical investigation subject administered a pharmaceutical product and that does not necessarily have a causal relationship with this treatment’.

An elective procedure not reflecting a worsening of a known underlying medical condition is not considered an AE, and therefore will not be considered an SAE despite requiring hospitalisation. However, complications of a procedure will be considered an AE and may be considered an SAE if hospitalisation is prolonged (or any other SAE criteria is met). A hospitalisation or prolongation of a hospitalisation for reasons other than an AE would not be considered an SAE.

AEs include any occurrences that are new in onset or aggravated in severity or frequency from the baseline condition, or abnormal results of diagnostic procedures, including laboratory test abnormalities. Concurrent medical conditions present at baseline that worsen will be considered as AEs. Lack of efficacy, aggravation, or relapse of current infection are not an AE in the study and therefore also not an SAE (except death).

Events will be reviewed and classified by the site PI. The relationship of the event to the study drug and whether the event is an expected event or not will be assessed using the listing of adverse effects contained in the summary of product characteristics for the antibiotics used.

The treating team has the primary responsibility for reviewing laboratory test results and determining whether an abnormal value in an individual study participant requires action. In general, abnormal laboratory results without clinical significance (based on clinical judgement) should not be recorded as AEs. However, laboratory value changes requiring therapy or adjustment in prior therapy are considered adverse. The investigators should liaise closely with the treating teams and remain aware of any such AEs.

SAE are defined as an AE that:

- is fatal
- is life threatening (places the participant at immediate risk of death)
- requires inpatient hospitalisation or prolongation of existing hospitalisation
- results in persistent or significant disability/incapacity
- is a congenital anomaly/birth defect
- other significant medical hazard

For criteria ‘AST or ALT > 3 × upper limit of normal (ULN) and TBL > 2 × ULN or prothrombin time and international normalised ratio (PT-INR) > 1.5, if PT-INR measured (potential Hy’s law)’, the case must be reported as an SAE (if baseline AST or ALT is ≤ ULN). If baseline AST or ALT is > ULN, the case that meets the following criteria must be reported as an SAE. AST or ALT > 3 × increase from baseline AST or ALT and TBL > 2 × increase from baseline TBL.

The following criteria will be used when assessing kidney injury:

- Grade 1: Creatinine > 1.5 to 2× baseline and < 350 μmol/L
- Grade 2: Creatinine > 2 to 3× baseline and < 350 μmol/L
- Grade 3: Creatinine > 3× baseline and/or > 350 μmol/L
- Grade 4: Dialysis (if previously not on dialysis)

Death within 30 days from time of randomisation is the primary outcome measure of the study, and death within 14 and 90 days are the secondary outcome measures. Given the variability of mortality associated with Gram-negative bacteraemia (approximately 5–12%), death itself cannot be considered an ‘unanticipated’ event.

If any member of the trial team becomes aware of an unexpected death or SAE at any stage of the trial, the PI will be alerted. The PI should report all deaths and SAEs to their local regulatory authority, and all deaths and AEs will be recorded and reported in the final analysis. Unforeseen AEs will be discussed with collaborating investigators at other centres; such information will be reviewed by regular teleconference.

Unanticipated Problems Involving Risk to Subjects or Others (UPIRTSO) events and SAEs are defined below. Events will be reviewed and classified by the site PI or other investigator. Severity will be classified using a standard set of criteria for grading AEs (Common Terminology Criteria for Adverse Events version 5.0). The relationship of the event to the study drug and whether the event is an expected event or not will be assessed using the listing of adverse effects contained in the summary of product characteristics for the antibiotics used.

Any events that are unexpected (in terms of severity or frequency) that can reasonably be attributed to the drug under study and that may expose other subjects to harm will be reported. UPIRTSO events refer to problems, in general, to include any incident, experience or outcome (including AEs) that meets ALL of the following criteria:

- Unexpected—in terms of nature, severity or frequency of the problem as described in the study documentation (e.g. Protocol, Consent documents)
- Related or possibly related to participation in the research—possibly related means there is a reasonable possibility that the problem may have been caused by the procedures involved in the research
- Risk of harm—this suggests that the research places participants or others at a greater risk of harm (including physical, psychological, economic or social harm) than was previously known or recognised

Urgent reporting timeline for UPIRTSO events to Singapore’s National Healthcare Group Domain Specific Review Board (NHG DSRB): All problems involving local deaths, whether related or not, should be reported immediately—within 24 h after first knowledge by the local PI.

Expedited reporting timeline for UPIRTSO events to NHG DSRB: All other problems must be reported as soon as possible but not later than 7 calendar days after first knowledge by the local investigator.

Medical and scientific judgement should be exercised in determining whether an event is an important medical event. An important medical event may not be immediately life threatening and/or result in death or hospitalisation. However, if it is determined that the event may jeopardise the subject and/or may require intervention to prevent one of the other AE outcomes, the important medical event should be reported as serious. All SAEs that are unexpected and related to the study drug will be reported to Singapore’s Health Sciences Authority (HSA). The investigator will be responsible for informing HSA no later than 15 calendar days after first knowledge that the case qualifies for expedited reporting. Follow-up information will be actively sought and submitted as it becomes available. For fatal or life-threatening cases, HSA will be notified as soon as possible but no later than 7 calendar days after first knowledge that a case qualifies, followed by a complete report within 8 additional calendar days.

### Frequency and plans for auditing trial conduct {23}

The study will be monitored by an independent monitoring team to ensure data quality and accuracy. Study monitoring will be provided by clinical research associates (CRAs) from the Clinical Research & Innovation Office (CRIO) of Tan Tock Seng Hospital as well as SCRN members who are not delegated to the trial. The CRAs will monitor in accordance with the CRIO’s monitoring plan and GCP to ensure high level of confidence in the integrity and quality of the data. The monitors will contact and visit each site PI at periodic intervals and will be allowed, on request, to inspect the various records (source documents, eCRFs and other pertinent data) provided subject confidentiality is maintained in accordance with local regulations. It will be the monitor’s responsibility to inspect the eCRFs throughout the study, to verify adherence to the protocol, and to ensure completeness, consistency and accuracy of the data being entered. The monitor will verify that the subject received the study drug assigned by the randomisation centre. The monitor will have access to laboratory test reports and other subject records needed to verify the entries on the eCRF. The site PIs agree to cooperate with the monitor to ensure that any problems detected in the course of these monitoring visits are resolved in a timely manner. If it is not possible to perform on-site monitoring visits due to travel restrictions or entry restrictions into hospitals due to COVID-19, key data fields (efficacy and safety data points) will be remotely monitored by the CRIO or SCRN representatives. Additionally, the study may be audited by regulatory authorities who must be allowed access to CRFs, source documents and other study files.

### Plans for communicating important protocol amendments to relevant parties (e.g. trial participants, ethical committees) {25}

The global project manager and the overall PI of the trial will be responsible for communicating important protocol amendments to relevant parties including the GTSC, DSMB and site PIs and co-investigators. The communications may be done via emails and/or teleconferences.

### Dissemination plans {31a}

The data obtained from all participating sites will be pooled and analysed together as soon as possible after trial completion. Individual researchers will not publish data from the trial until the main study publication has been released. The site PIs and GSTC will form the main writing committee to communicate the trial results to the public.

## Discussion

This study aims to determine whether patients with uncomplicated Gram-negative bacteraemia can step down to oral antibiotic treatment as early as within 72 h from the time of index blood culture collection. Recent data from a multicentre, propensity score-matched, retrospective study suggested 7 days of antibiotics may be sufficient for treatment of uncomplicated *Enterobacterales* bacteraemia [23]. Consistent with this, recently completed RCTs demonstrated non-inferiority of 7 days versus 14 days of antibiotic treatment for uncomplicated Gram-negative bacteraemia [20, 24]. Therefore, switching to oral therapy on day 7 or later will not be meaningful as it is likely that sufficient IV antibiotics would have already been administered. In this study, we propose 72 h from the time of index blood culture collection as the cut-off time frame for switching to oral therapy for patients randomised to the intervention arm. The proposed 72-h randomisation window is supported by the observational analyses of Rieger et al., Kutob et al. and Mercuro et al., where patients typically received 3–5 days of IV therapy prior to oral stepdown therapy [4, 16, 17].

The options for oral therapy in this study are fluoroquinolones or trimethoprim-sulfamethoxazole—selected due to their extensive use in clinical practice with well-established safety and efficacy across a plethora of infections. For example, in one of the aforementioned RCTs studying treatment duration for uncomplicated Gram-negative bacteraemia, >70% (across both study arms) received oral fluoroquinolones among the patients who switched to stepdown therapy [20].

Our study will help inform local and international practice guidelines on optimal antibiotic management for uncomplicated Gram-negative bacteraemia. A finding of non-inferiority in clinical efficacy of oral fluoroquinolones or trimethoprim-sulfamethoxazole versus IV antibiotics may translate to wider adoption of a more cost-effective treatment strategy. Early oral stepdown therapy will reduce hospital LOS as well as result in better patient-centred quality of life outcomes and satisfaction.

## Trial status

Participant recruitment opened first at the Tan Tock Seng Hospital site in May 2022. The other participating sites will open recruitment progressively from the third quarter of 2022 onwards. Recruitment is expected to be completed by the end of 2024 or first quarter of 2025.

## Supplementary Information

**Additional file 1.**

## Data Availability

The PI and his designee (e.g. data management team, statistical team) will have access to the final trial dataset. The PI will keep any records, study files or source documentation for a minimum of 15 years after the completion of the trial before being destroyed or erased. These documents may be retained for a longer period if required by the applicable regulatory requirements or institutional policy. Any data required to support the protocol can be supplied on request.

## Abbreviations

AE: Adverse event
ALP: Alkaline phosphatase
ALT: Alanine transaminase
AST: Aspartate transaminase
CRA: Clinical research associate
CRF: Case report form
CRIO: Clinical Research & Innovation Office
CRP: C-reactive protein
DOOR: Desirability Of Outcome Ranking
DSMB: Data Safety Monitoring Board
EC: Ethics Committee
eCRF: Electronic case report form
ESBL: Extended-spectrum beta-lactamase
FBC: Full blood count
FDA: Food and Drug Administration
GNB: Gram-negative bacteria
GTSC: Global Trial Steering Committee
HSA: Health Sciences Authority
ICER: Incremental cost-effectiveness ratio
ICF: Informed consent form
ICH-GCP: International Conference on Harmonisation-Good Clinical Practice
ICU: Intensive care unit
IRB: Institutional Review Board
ITT: Intention-to-treat
IV: Intravenous
LOS: Length of stay
mIIT: Modified intention-to-treat
NHG DSRB: National Healthcare Group Domain Specific Review Board
OPAT: Outpatient parenteral antibiotic therapy
PI: Principal Investigator
PK/PD: Pharmacokinetics/pharmacodynamics
PT-INR: Prothrombin time and international normalised ratio
RCT: Randomised controlled trial
SAE: Serious adverse event
SCRN: Singapore ID Clinical Research Network
SOP: Standard operating procedures
TBL: Total bilirubin
UPIRSO: Unanticipated Problems Involving Risk to Subjects or Others
ULN: Upper limit of normal
