# Supplementary material for: Early oral stepdown antibiotic therapy versus continuing intravenous therapy for uncomplicated Gram-negative bacteraemia (the INVEST trial): study protocol for a multicentre, randomised controlled, open-label, phase III, non-inferiority trial
Source: Trials. 2022 Jul 19;23:572. doi: 10.1186/s13063-022-06495-3 (PMC9295110; doi:10.1186/s13063-022-06495-3)
Supplement: Supplementary file 1 — Additional file 1. [file 13063_2022_6495_MOESM1_ESM.zip › INVEST_Continuing ICF_English_V6.0_27May2022R1.doc]

**INFORMED CONSENT FORM**

**1. Study Information**

**Protocol Title:**

Early oral step-down ant**i**biotic therapy versus co**n**tinuing intra**ve**nou**s** **t**herapy for uncomplicated Gram-negative bacteraemia (the INVEST trial)

**Site Principal Investigators & Contact Details**

| **A/Prof David Lye**  National Centre for Infectious Diseases  Tan Tock Seng Hospital  Tel: 6357 7457 (office) / 9006 6785 (mobile)  Email: [David_Lye@ttsh.com.sg](mailto:David_Lye@ttsh.com.sg)  **Dr Jenna Chaung**  Division of Infectious Diseases  Ng Teng Fong General Hospital  Tel: 8118 6590 (mobile)  Email: [jenna_chaung@nuhs.edu.sg](mailto:jenna_chaung@nuhs.edu.sg) | **A/Prof Sophia Archuleta**  Division of Infectious Diseases  National University Hospital  Tel: 6772 2002 (office) /  8186 9626 (mobile)  Email: [sophia@nus.edu.sg](mailto:sophia@nus.edu.sg) |
| --- | --- |
|  |  |

**2. Purpose of the Research Study**

*You have been enrolled in this study in accordance with Singapore law which allows the participant’s legal representative to consent to the participant taking part in a study when the participant is unable to provide consent for himself/herself. Your legal representative had consented to your participation in this study at a time when you were unable to consent for yourself. You are now invited to consider whether you wish to continue taking part in this study.*

You were enrolled because you have been diagnosed with bloodstream infection caused by Gram-negative bacteria. Gram-negative bacteria are common causes of infection in your bloodstream, particularly in people who are hospitalised or receiving treatment for a medical condition in the community. Patients with bloodstream infections due to Gram-negative bacteria can be treated with a number of antibiotics in different dosage forms (for example, oral form for consumption through your mouth or intravenous form via injection through your veins). You have been judged by the doctor to be clinically stable and not critically ill, and your bloodstream infection has been assessed to be uncomplicated. In such a clinical scenario, the typical regimen would consist of 1–2 weeks of antibiotic treatment. One area that is unclear in patients with uncomplicated bloodstream infections caused by Gram-negative bacteria is whether they can “step down” early to oral antibiotics instead of undergoing prolong treatment with intravenous antibiotics. The use of oral antibiotics has many advantages over the use of intravenous antibiotics including lower healthcare cost, greater convenience, and elimination of pain, discomfort or risk of adverse events associated with needle use.

The purpose of this study is to determine whether oral antibiotics are as effective and safe compared with intravenous antibiotics in treating uncomplicated bloodstream infections by Gram-negative bacteria. You were randomly assigned into either the standard arm group or the intervention arm group. In the standard arm group, patients were given intravenous antibiotics for at least another 24 hours post randomisation. In the intervention arm group, patients were immediately stepped down to oral antibiotics. Regardless of whether you were assigned to the standard arm or intervention arm groups, an effective antibiotic against the Gram-negative bacteria in your body were given to you. The Gram-negative bacteria isolated from your body was tested against many different antibiotics in the hospital microbiology laboratory, and only effective antibiotics that killed the bacteria in the laboratory were given to you. Therefore, the main difference between the standard arm and intervention arm groups was the route of antibiotic administration. This Phase III clinical research study does not involve the testing of any new drugs. The study has been designed to ensure the researchers and doctors interpret the results in a fair manner and do not jump to conclusions.

A total of approximately 720 patients (360 participants on standard arm and 360 participants on intervention arm) will participate in this study from various approved sites in Singapore, Australia, England, Malaysia and South Korea. Recruitment will take place in Tan Tock Seng Hospital, National University Hospital, Singapore General Hospital, Changi General Hospital, Ng Teng Fong General Hospital and Sengkang General Hospital, where approximately 480 participants are expected to be enrolled between the first quarter of 2022 and the last quarter of 2024.

**3. What procedures will be followed in this study**

You have bloodstream infection with Gram-negative bacteria confirmed through at least one positive blood culture test. A research doctor, nurse or coordinator will discuss with you the following procedures, a few of which may be additional to the routine medical care you would normally receive.

Agreeing to continue to participate in this study means that you are willing to partake in the following procedures:

a) Randomisation to standard arm group which means you will receive the standard-of-care intravenous antibiotic treatment your doctor would normally use for this medical condition, or randomisation to intervention arm group which means you will receive an early “step down” to oral antibiotic treatment. Randomisation means assigning you into one of the two groups with a 50:50 chance – like tossing a coin. If you are assigned to the standard arm group, the intravenous antibiotics may be given to you as an inpatient or as outpatient parenteral antibiotic therapy. If you are assigned to the intervention arm group, the oral antibiotics may be given to you as an inpatient or you may be discharged from hospital and asked to consume the oral antibiotics at home. The treatment duration for standard arm and intervention arm groups will be determined by your treating doctor, which is usually around 1 week.
b) Blood tests will be taken over the course of your involvement in the study by a doctor, nurse, phlebotomist, or research coordinator. Blood tests are part of routine clinical care. For example, a blood test for laboratory investigations may be used to examine your kidney and liver functions as well as to determine your full blood count and C-reactive protein (inflammatory biomarker) level. For this research study, you will be requested to provide approximately 10 mL (2 teaspoons) of blood samples for laboratory investigations at two times points: day of screening (baseline) and day of hospital discharge. If your treating doctor orders these routine blood tests within 24 hours of the above-mentioned time points, there is no need for additional blood collection. The blood obtained will be analysed only for the purpose of this study and it will not be stored for this research or for future research. No incidental findings are expected to arise from the blood tests as they are standard clinical laboratory investigations routinely ordered by hospital doctors and they do not involve any genetic testing. Incidental findings are findings that have potential health or reproductive importance to research participants like you/your child and are discovered in the course of conducting the study, but are unrelated to the purposes, objectives or variables of the study. These findings may affect your current or future life and/or health insurance coverage. There will not be any incidental findings arising from this research. You may be asked to perform a urine pregnancy test at screening, as pregnancy is an exclusion criterion for this study (note: this is likely to be irrelevant to you at this stage, as this is a consent form for continuing participation in the study).
c) Information about your demographics (e.g. age, gender), medical history (e.g. underlying diseases, antibiotic history) and healthcare expenses will be obtained from you or from your medical records or from administrative sources. You may be asked to retain the medical bills/receipts for this treatment episode. Your compliance to the antibiotic treatment, your body’s response to the antibiotic treatment (e.g. adverse events, health status) as well as relevant clinical/laboratory information will also be monitored and recorded during the entire duration of the study. We are also interested in finding out your quality of life during this infection episode and will ask you to complete a quality of life survey at 3 times points: day of screening after informed consent taking, final day of study drug treatment, and at approximately study day 90. Overall, from the day you get discharged until the end of the study period, you can expect to receive a maximum of 4 telephone calls OR 3 telephone calls and 1 home visit OR 2 telephone calls and 2 home visits from the study team (approximately on the final day of study drug treatment, study days 14, 30 and 90) for the above-mentioned purposes.

**4. Your Responsibilities in This Study**

If you agree to continue to participate in this study, you should follow the advice given to you by the study team. You should adhere to the study drug treatment given to you (e.g. not forget to consume your oral medications if you are placed in the intervention arm group), and the study team will conduct an adherence check at the end of the study drug treatment. You should be prepared to undergo the blood tests and complete the quality of life surveys and all the study procedures outlined in Section 3 of this consent form. In particular, you should be prepared to receive a few telephone calls OR home visits from the study team after your discharge from the hospital, primarily for the purpose of assessing your well-being up to study day 90.

**5. What Is Experimental in This Study**

Early step down (within 72 hours from the time of your positive blood culture) to oral antibiotics for treatment of bloodstream infection is experimental in this study. If you were assigned to the intervention arm, you were prescribed with oral fluoroquinolones (most commonly, ciprofloxacin) or oral trimethoprim-sulfamethoxazole. Before being assessed to be eligible for this study, we verified with the hospital microbiology laboratory that the oral antibiotics used in this study effectively killed the Gram-negative bacteria isolated from your blood. If you were assigned to the standard arm, you were likewise given an effective intravenous antibiotic as per standard hospital practice. A commonly used intravenous antibiotic for your condition is ceftriaxone.

**6. Possible Risks and Side Effects**

a) Any antibiotic treatment regimen, whether it is orally administered or intravenously administered, has a risk of treatment failure. While the study team hypothesises that oral antibiotics in intervention arm is clinically non-inferior to intravenous antibiotics in standard arm, there is a possibility that risk of treatment failure may be higher in intervention arm compared with standard arm. Your doctor will be monitoring you closely for possible treatment failure associated with the antibiotics being administered to you. In the event of treatment failure, the doctor may change the treatment strategy at any time point post-randomisation. This may include, but are not limited to, switching to an intravenous antibiotic from the original oral antibiotic regimen, or switching to a more broad-spectrum intravenous antibiotic from the original intravenous antibiotic regimen.
b) Any antibiotic treatment can cause side effects. You may have none, some or all of the side effects listed below, and they may be mild, moderate or severe. Many side effects go away shortly after the treatment ends. However, side effects can sometimes be serious, long lasting or permanent. If a severe side effect or reaction occurs, your doctor may need to stop your treatment. Your doctor will discuss the best way of managing any side effects with you.

Ciprofloxacin – Nausea (3%); diarrhoea (2%); vomiting (1%); rash (1%); and rarely (<1%) disturbance of liver, kidney or heart function, seizures, high or low blood sugar, or tendon rupture.

Trimethoprim-sulfamethoxazole – The most common adverse reactions include gastrointestinal upset (nausea, vomiting, anorexia) and dermatologic reactions (rash or urticaria). Rare, life-threatening reactions have been associated with trimethoprim-sulfamethoxazole, including severe skin rashes, abnormalities of blood counts, and liver damage.

Ceftriaxone – Rash (2%); diarrhoea (3%); abnormal blood counts (2–6%); abnormal liver tests (3%); abnormal kidney tests (1%)

c) Having blood taken or being administered intravenous antibiotics may cause some discomfort, bruising or bleeding at the site of the needle stick and rarely an infection will occur. Although these may happen, they can be readily treated.

**7. Possible Benefits from Participating in the Study**

All participants will be given the same high level of medical care that is routinely given to them by their hospital doctors. You may or may not personally receive a benefit from participating in this study but the medical information gain from the study may benefit other patients in the future. For example, if this study finds oral antibiotics to be as effective as intravenous antibiotics, future patients with uncomplicated bloodstream infections by Gram-negative bacteria may not be placed on intravenous catheters for prolonged periods and this will reduce their hospital length of stay resulting in healthcare cost savings and greater convenience. Similarly, if you were randomised to intervention arm, you may enjoy better quality of life (e.g. earlier discharge from hospital, elimination of pain and discomfort associated with needle use) compared with participants in standard arm.

**8. Important Information for Women Subjects**

It is important that you are not pregnant or breast-feeding, and do not become pregnant during the course of your antibiotic treatment. This is because there is a wide range of antibiotic options available, particularly in the standard arm, and the effects of some of these antibiotics on your baby are unknown. If you are pregnant or trying to become pregnant or breast feeding, you should not join this study.

**9. Alternatives to Participation**

You do not have to continue to take part in this research study to receive treatment in this hospital. Other options are available, including continuing on with the standard of care intravenous antibiotic treatment recommended by your treating team. The study team doctor will discuss these options with you before you decide whether or not to take part in this research study.

**10. Costs & Payments if Participating in the Study**

There are no additional costs associated with participating in this research study. All blood tests for laboratory investigations and urine pregnancy test (if applicable) required as part of the research study will be provided to you free of charge.

**11. Voluntary Participation**

Taking part in any research study is voluntary. If you do not wish to continue to take part, you are not obliged to. If you decide to continue to take part and later change your mind, you are free to withdraw from the study at any stage by notifying a study team member. Your decision to continue to take part or not, or to take part and later withdraw, will not affect your medical care or any benefits you are entitled to or your relationship with the treating and study teams. If you choose to withdraw from the study at any stage, the information that has been collected for the study matter up until that point will be used as part of the data analysis for the study. The reason is to enable a complete and comprehensive evaluation of the study.

Before you make your decision, a study team member will be available to answer any questions you might have about the research. You may also wish to discuss the study with your treating doctor or a family member. Sign the consent form only after you have had a chance to ask questions and have received satisfactory answers.

Your treating doctor or the site Principal Investigator of this study may stop your study participation at any time if they decide it is in your best interests. They may also do this if you do not follow the instructions required to complete the study adequately. If you are later found to have a more serious medical problem (e.g. uncontrolled focus of infection like brain abscess) or severe side effects, your doctors will decide if you may continue to participate in the study. New information about risks and benefits may become known to the researchers over the course of the study. When this occurs, you will be informed right away especially if it concerns your willingness to continue in the study. This new information may mean that you can no longer participate in the research. Under all circumstances, you will be offered all available clinical care to suit your needs and medical condition.

The biological samples collected for the study will be deemed to be gifted to Tan Tock Seng Hospital, National University Hospital or Ng Teng Fong General Hospital and will not be returned to you. You will also not have any right or claim to any share in the commercial gain derived from the research (if any). However, you retain your right to ask the Principal Investigator to discard or destroy any remaining samples if the biological sample(s) is individually-identifiable and has not been used for the research or it has been used for research but it is practicable to discontinue further use of the biological sample(s) for the research.

**12. Compensation for Injury**

If you follow the directions of the doctors in charge of this study and you are physically harmed due to the antibiotics or procedures given under the study plan, the hospital’s clinical trial compensation scheme will pay the medical expenses for the treatment of that injury. Tan Tock Seng Hospital, National University Hospital or Ng Teng Fong General Hospital without legal commitment will compensate you for the injuries arising from your study participation without you having to prove the hospital is at fault. There are however conditions and limitations to the extent of compensation provided. You may wish to discuss this with the site Principal Investigator. By signing this consent form, you will not waive any of your legal rights or release the parties involved in this study from liability for negligence.

**13. Confidentiality of Study and Medical Records**

Your continued participation in this study will involve the collection of “Personal Data”. “Personal Data” means data about you which makes you identifiable (i) from such data or (ii) from that data and other information which an organisation has or likely to have access. This includes medical conditions, medications, investigations and treatment history. Any biological samples and/or information containing your “Personal Data” that is collected for the purposes described in this Informed Consent Form will not be transferred out of Singapore. By signing the consent form, you agree to the study team doctor and relevant research staffs collecting and using personal information about you for the research study. Any information obtained in connection with this research study that can identify you will remain confidential. Your hospital will use your personal information as required for your care, to collect accurate data for the study, and if necessary to contact you about the study. If you withdraw your consent to take part in the study, your hospital will not collect any further information about you for the purpose of the study but will keep the information already collected solely for the purpose of the study. To safeguard your rights, your hospital will collect and use as little personal information as possible. Data collected and entered into the Case Report Forms are the property of Tan Tock Seng Hospital, National University Hospital or Ng Teng Fong General Hospital. In the event of any publication regarding this study, your identity will remain confidential. Your data will be stored in National Healthcare Group (NHG) REDCap platform or an alternative platform that complies with IHiS security recommendation. Your personal information will never be made accessible to anyone outside of this research study team. Any individually-identifiable data obtained during the course of this study will be stored and analysed for the purposes of this study and will not be used for future biomedical research.

Your health records and any information obtained during the research study are subject to inspection for the purpose of verifying the procedures and data by the relevant authorities (NHG Domain Specific Review Board, Ministry of Health, Health Sciences Authority), the institution relevant to this participant information sheet (Tan Tock Seng Hospital, National University Health System [National University Hospital], Ng Teng Fong General Hospital), or as required by law. In addition, research arising in the future, based on your “Personal Data”, will be subject to review by the relevant Institutional Review Board. By signing the consent form, you authorise release of, or access to, this confidential information to the relevant study personnel and regulatory authorities as noted. At all times, all of the people involved in this study will have an obligation to keep the information about you as a research participant confidential and your data will be protected as confidential by law. If any information about this study is published or presented, your identity will remain confidential.

By participating in this research study, you are confirming that you have read, understood and consent to the Personal Data Protection Notification available at:

Tan Tock Seng Hospital

<https://www.ttsh.com.sg/Patients-and-Visitors/Your-Hospital-Stay/Pages/Patients-Rights.aspx>

National University Health System (National University Hospital)

<https://www.nuh.com.sg/Pages/Personal-Data-Protection-Act.aspx>

Ng Teng Fong General Hospital

<https://www.ntfgh.com.sg/Pages/Personal-Data-Protection-Act.aspx>

Hard copies are also available on request.

**14. Storage of bacterial isolates for future research**

This study examines the efficacy and safety of different antibiotics for treatment of uncomplicated bloodstream infections by Gram-negative bacteria. To enhance our understanding of how patients respond to these treatments, further laboratory study of the bacteria isolated from your blood may be necessary. The bacteria will be stored in the hospital microbiology laboratory as per standard institutional practice. We may examine the mechanisms that lead to antibiotic resistance or investigate what makes the bacteria harmful to humans. No blood samples will be stored for other research purposes; only the bacteria isolated from your blood samples. The stored bacterial isolates will not contain any identifiable information that can be directly linked to you (that is, it will be coded or de-identified). This aspect of the research will not have any direct impact on you or your current treatment.

**15. Who To Contact if You Have Questions**

If you have questions about this research study or in case of any injuries during the course of this study, you may contact the site Principal Investigators:

| **A/Prof David Lye**  National Centre for Infectious Diseases  Tan Tock Seng Hospital  Tel: 6357 7457 (office) / 9006 6785 (mobile)  Email: [David_Lye@ttsh.com.sg](mailto:David_Lye@ttsh.com.sg)  **Dr Jenna Chaung**  Division of Infectious Diseases  Ng Teng Fong General Hospital  Tel: 8118 6590 (mobile)  Email: [jenna_chaung@nuhs.edu.sg](mailto:jenna_chaung@nuhs.edu.sg) | **A/Prof Sophia Archuleta**  Division of Infectious Diseases  National University Hospital  Tel: 6772 2002 (office) /  8186 9626 (mobile)  Email: [sophia@nus.edu.sg](mailto:sophia@nus.edu.sg) |
| --- | --- |

The study has been reviewed by the NHG Domain Specific Review Board for ethics approval. If you want an independent opinion of your rights as a research subject, you may contact the NHG Domain Specific Review Board Secretariat at 6471-3266. You can also find more information about the NHG Domain Specific Review Board at [www.research.nhg.com.sg](http://www.research.nhg.com.sg/).

If you have any complaints about this research study, you may contact the site Principal Investigator or the NHG Domain Specific Review Board Secretariat.

#### **CONSENT FORM**

| **Protocol Title:** | | | |
| --- | --- | --- | --- |
| Early oral step-down ant**i**biotic therapy versus co**n**tinuing intra**ve**nou**s** **t**herapy for uncomplicated Gram-negative bacteraemia (the INVEST trial) | | | |
| **Site Principal Investigators & Contact Details** | | | |
|  | **A/Prof David Lye**  National Centre for Infectious Diseases  Tan Tock Seng Hospital  Tel: 6357 7457 (office) / 9006 6785 (mobile)  Email: [David_Lye@ttsh.com.sg](mailto:David_Lye@ttsh.com.sg)  **Dr Jenna Chaung**  Division of Infectious Diseases  Ng Teng Fong General Hospital  Tel: 8118 6590 (mobile)  Email: [jenna_chaung@nuhs.edu.sg](mailto:jenna_chaung@nuhs.edu.sg) | **A/Prof Sophia Archuleta**  Division of Infectious Diseases  National University Hospital  Tel: 6772 2002 (office) /  8186 9626 (mobile)  Email: [sophia@nus.edu.sg](mailto:sophia@nus.edu.sg) |  |

I voluntarily consent to continue to take part in this research study. I have fully discussed and understood the purpose and procedures of this study. This study has been explained to me in a language that I understand. I have been given enough time to ask any questions that I have about the study, and all my questions have been answered to my satisfaction.

By participating in this research study, I confirm that I have read, understood and consent to the Tan Tock Seng Hospital / National University Hospital’s Data Protection Policy. I also consent to the use of my Personal Data for the purposes of engaging in related research arising in the future.

I also give permission for information in my medical records to be used for research. In any event of publication, I understand that this information will not bear my name or other identifiers and that due care will be taken to preserve the confidentiality of this information.

Name of Participant	Signature	Date

_______________________

Time (HH:MM)

**Translator Information (If Applicable)**

The study has been explained to the participant in

*______________* by *_____________* _______	_____________________

Language	Name of Translator	Designation

##### Witness Statement (If Applicable)

I, the undersigned, certify to the best of my knowledge that the participant signing this informed consent form had the study fully explained in a language understood by him / her and clearly understands the nature, risks and benefits of his / her participation in the study.

Name of Witness	Signature	Date

_______________________

Time (HH:MM)

##### Investigator Statement

I, the undersigned, certify that I explained the study to the participant and to the best of my knowledge the participant signing this informed consent form clearly understands the nature, risks and benefits of her participation in the study.

Name of Investigator	Signature	Date

administering consent

_______________________

Time (HH:MM)
